# Anti-SARS-CoV-2 receptor-binding domain antibody evolution after mRNA vaccination

**DOI:** 10.1038/s41586-021-04060-7

**Published:** 2021-10-07

**Authors:** Alice Cho, Frauke Muecksch, Dennis Schaefer-Babajew, Zijun Wang, Shlomo Finkin, Christian Gaebler, Victor Ramos, Melissa Cipolla, Pilar Mendoza, Marianna Agudelo, Eva Bednarski, Justin DaSilva, Irina Shimeliovich, Juan Dizon, Mridushi Daga, Katrina G. Millard, Martina Turroja, Fabian Schmidt, Fengwen Zhang, Tarek Ben Tanfous, Mila Jankovic, Thiago Y. Oliveria, Anna Gazumyan, Marina Caskey, Paul D. Bieniasz, Theodora Hatziioannou, Michel C. Nussenzweig

**Affiliations:** 1 Laboratory of Molecular Immunology, The Rockefeller University, New York, NY USA; 2 Laboratory of Retrovirology, The Rockefeller University, New York, NY USA; 3 Howard Hughes Medical Institute, New York, NY USA

**Keywords:** Antibodies, Vaccines, SARS-CoV-2

## Abstract

Severe acute respiratory syndrome coronavirus 2 (SARS-CoV-2) infection produces B cell responses that continue to evolve for at least a year. During that time, memory B cells express increasingly broad and potent antibodies that are resistant to mutations found in variants of concern^1^. As a result, vaccination of coronavirus disease 2019 (COVID-19) convalescent individuals with currently available mRNA vaccines produces high levels of plasma neutralizing activity against all variants tested^1,2^. Here we examine memory B cell evolution five months after vaccination with either Moderna (mRNA-1273) or Pfizer-BioNTech (BNT162b2) mRNA vaccine in a cohort of SARS-CoV-2-naive individuals. Between prime and boost, memory B cells produce antibodies that evolve increased neutralizing activity, but there is no further increase in potency or breadth thereafter. Instead, memory B cells that emerge five months after vaccination of naive individuals express antibodies that are similar to those that dominate the initial response. While individual memory antibodies selected over time by natural infection have greater potency and breadth than antibodies elicited by vaccination, the overall neutralizing potency of plasma is greater following vaccination. These results suggest that boosting vaccinated individuals with currently available mRNA vaccines will increase plasma neutralizing activity but may not produce antibodies with equivalent breadth to those obtained by vaccinating convalescent individuals.

## Abstract (web summary)

Individual memory antibodies selected over time by natural infection with SARS-CoV-2 have greater potency and breadth than antibodies elicited by vaccination, whereas the overall neutralizing potency of plasma is greater following vaccination.

## Main

Between 21 January and 20 July 2021, we recruited 32 volunteers with no history of prior SARS-CoV-2 infection receiving either Moderna (mRNA-1273; *n* = 8) or Pfizer-BioNTech (BNT162b2; *n* = 24) mRNA vaccine for sequential blood donation. Matched samples were obtained at two or three time points. Individuals indicated as ‘prime’ were sampled an average of 2.5 weeks after receiving their first vaccine dose. Individuals who completed their vaccination regimen were sampled an average of 1.3 months after the boost (median = 35.5 days), which is not statistically different from the sampling at 1.3 months in our naturally infected cohort^3^ (median = 38.5 days, *P* = 0.21). Individuals sampled at 1.3 months were sampled again approximately 5 months after the second vaccine dose. The volunteers ranged in age from 23 to 78 years old (median = 34.5 years old), 53% were male and 47% were female (for details, see Methods and Supplementary Tables 1 and 2).

## Plasma binding and neutralization assays

**Fig. 1:**
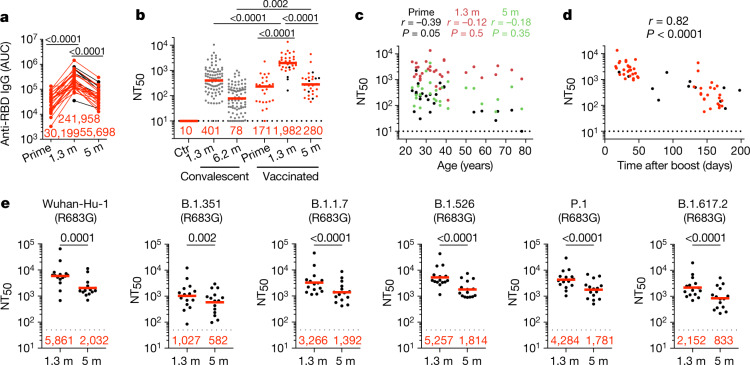
Plasma ELISAs and neutralizing activity. **a**, Graph showing area under the curve (AUC) for plasma IgG binding to SARS-CoV-2 RBD after prime and 1.3 and 5 months (m) after the second vaccine dose for *n* = 32 paired samples. Samples without a prime value are shown in black. **b**, NT_50_ values in plasma from pre-pandemic controls (Ctr, *n* = 3), convalescent individuals 1.3 months (ref. ^3^) and 6.2 months (ref. ^7^) after infection (grey), and vaccinated individuals (*n* = 32) after prime and 1.3 and 5 months after receiving two doses of mRNA vaccine. Samples without a prime value are shown in black. **c**, NT_50_ values (*y* axis) versus age (*x* axis) in *n* = 32 individuals after prime (black) and 1.3 months (orange) or 5 months (green) after boosting with an mRNA vaccine. **d**, Graph showing NT_50_ values (*y* axis) versus days after boost (*x* axis) in *n* = 32 individuals receiving two doses of an mRNA vaccine. Samples without a prime value are shown in black. **e**, Plasma neutralizing activity against the indicated SARS-CoV-2 variants of interest/concern (*n* = 15 paired samples at 1.3 and 5 months after full vaccination). Refer to the Methods for a list of all substitutions, deletions and insertions in the spike variants. All experiments were performed at least in duplicate. Red bars and values in **a**, **b** and **e** represent geometric mean values. Statistical significance in **a**, **b** and **e** was determined by two-tailed Kruskal–Wallis test with subsequent Dunn’s multiple-comparisons test and in **c** and **d** was determined by two-tailed Spearman correlation test.

**Extended Data Fig. 1:**
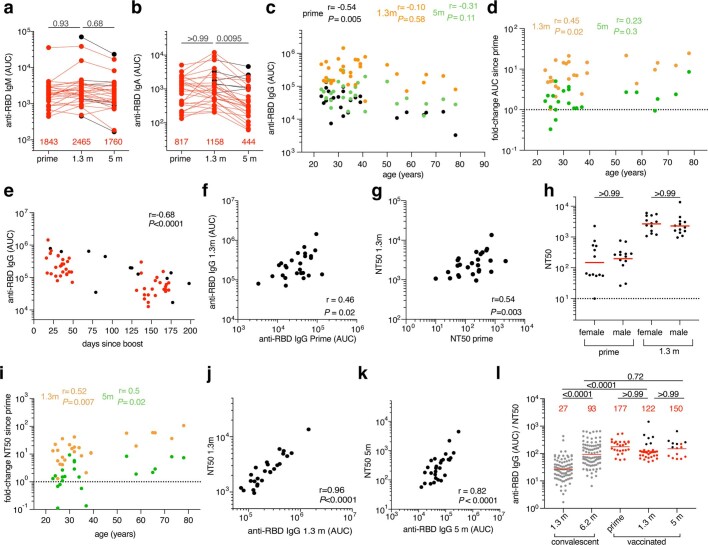
Plasma ELISA and neutralization. **a,b**, Graph shows area under the curve (AUC, Y-axis) for plasma IgM (**a**) or IgA (**b**) antibody binding to SARS-CoV-2 RBD after prime, and 1.3- and 5-months post-boost for paired samples from n=32 vaccinated individuals. Samples without a prime value are shown in black. **c**, Graph shows plasma IgG antibody binding (AUC, Y-axis) plotted against age (X-axis) after prime (black), and 1.3 months (orange) and 5 months (green) post-second vaccination in n=32 vaccinated individuals. **d**, Graph shows age (years, X-axis) vs. fold-change of IgG-binding titers (AUC, Y-Axis) between prime and 1.3m (orange) or 5m (green) post-boost in n=32 vaccinated individuals. **e**, Graph shows plasma IgG antibody binding AUC values (Y-axis) plotted against time after vaccination (day, X-axis) from n=32 vaccinated individuals. Samples without a prime value are shown in black. **f**, IgG antibody binding after prime (AUC, X-axis) vs. IgG antibody binding after 1.3 months post-boost (AUC, Y-axis) (n=26). **g**, NT50 values after prime (X-axis) vs. NT50 values after 1.3 months post-boost (Y-axis) in individuals receiving two doses of an mRNA vaccine (n=26). **h**, NT50 values after prime and 1.3 months post-boost in females and males receiving 2 doses of an mRNA vaccine (n=26).**i**, Graph shows age (years, X-axis) vs fold-change of NT50 (X-axis) between prime and 1.3m (orange) or 5m (green) post-boost (n=26). **j**, NT50 values (Y-axis) vs. IgG antibody binding (AUC, X-axis) 1.3 months after 2 doses of an mRNA vaccine (n=26). **k**, NT50 values (Y-axis) vs. IgG antibody binding (AUC, X-axis) 5 months after boost in individuals receiving two doses of an mRNA vaccine (n=28). **l**, Ratio of anti-RBD IgG antibody (AUC) to NT50 values (Y-axis) plotted for convalescent infected individuals (grey) 1.3m^3^ or 6.2m^7^ after infection, and from n=32 vaccinated individuals after the prime, and 1.3m and 5m after receiving 2 doses of an mRNA vaccine. Samples without a prime value are shown in black. All experiments were performed at least in duplicate. Red values or bar in **a**, **b, h** and **l** represent geometric mean values. Statistical significance in **a, b, h**, and **l** was determined by two-tailed Kruskal-Wallis test with subsequent Dunn’s multiple comparisons, or by two-tailed Spearman correlation test in **c, d, e, f, g, i, j**, and **k**.

Plasma IgM, IgG and IgA responses to SARS-CoV-2 receptor-binding domain (RBD) were measured by enzyme-linked immunosorbent assay (ELISA)^3^. As previously reported by others^2,4–6^, there was a significant increase in IgG reactivity to RBD between prime and boost (*P* < 0.0001) (Fig. 1a). IgM and IgA titres were lower than IgG titres and remained low after the second vaccine dose (Extended Data Fig. 1a, b). The magnitude of the response was inversely correlated with age after the prime (*r* = −0.54, *P* = 0.005), but in this limited sample set the age difference was no longer significant at 1.3 or 5 months after the second vaccine dose (Extended Data Fig. 1c, d). Between 1.3 and 5 months after the boost, anti-RBD titres of IgG and IgA decreased significantly. IgG titres decreased by an average of 4.3-fold (range, 1.7- to 10.2-fold), and the loss of activity was directly correlated with the time after vaccination (*P* < 0.0001) (Fig. 1a and Extended Data Fig. 1a, b, e).

Neutralizing activity was measured using HIV-1 pseudotyped with the SARS-CoV-2 spike (S) protein^1,3,7,8^. Naive individuals showed variable responses to the initial vaccine dose, with a geometric mean half-maximal neutralizing titre (NT_50_) of 171 (Fig. 1b and Supplementary Table 2). The magnitude of the neutralizing responses to the initial vaccine dose in naive volunteers was inversely correlated with age (*r* = −0.39, *P* = 0.05) (Fig. 1c). Both binding and neutralizing responses to the second vaccine dose were correlated with the prime responses (binding: *r* = 0.46, *P* = 0.02 (Extended Data Fig. 1f); neutralizing: *r* = 0.54, *P* = 0.003 (Extended Data Fig. 1g)), and there was a nearly 12-fold increase in the geometric mean neutralizing response that was similar in men and women with the age-related difference in neutralizing activity eliminated in the individuals in this cohort (Fig. 1b, c, Extended Data Fig. 1h, i). At 1.3 and 5 months after the boost, naive vaccinated individuals had 4.9- and 3.6-fold-higher neutralizing titres, respectively, than seen in a cohort of infected individuals measured at 1.3 months (ref. ^3^) and 6.2 months (ref. ^7^) after symptom onset (*P* < 0.0001) (Fig. 1b). Neutralizing responses were directly correlated with anti-RBD IgG titres (*r* = 0.96, *P* < 0.0001) (Extended Data Fig. 1j). Thus, the data obtained from this cohort agree with previous observations showing a significant increase in plasma neutralizing activity that is correlated with improved vaccine efficacy in naive individuals who receive the second dose of mRNA vaccine^2,6,9,10^ and higher neutralizing titres in fully vaccinated than in infected individuals^2,6^.

The 28 individuals assayed 5 months after vaccination had a 7.1-fold decrease in geometric mean neutralizing titre relative to their measurement at 1.3 months (*P* < 0.0001) (Fig. 1b), with a range of 1.4- to 27-fold decrease. Neutralizing activity was inversely correlated with the time from vaccination (*r* = −0.82, *P* < 0.0001) (Fig. 1d) and directly correlated with anti-RBD IgG binding titres assessed 5 months after vaccination (Extended Data Fig. 1k). As previously reported by others^11^, the ratio of binding to neutralizing serum titres was significantly higher in vaccinated individuals than in convalescent individuals at the 1.3-month time point (*P* < 0.0001) (Extended Data Fig. 1l). However, a difference was no longer apparent at the later time point (Extended Data Fig. 1l).

It has previously been shown that the neutralizing responses elicited by mRNA vaccination are more potent against the original Wuhan Hu-1 strain than they are against some of the currently circulating variants of concern^2,12–14^. To confirm these observations, we measured the neutralizing activity of 15 paired plasma samples obtained from naive individuals 1.3 and 5 months after the second vaccine dose against B.1.1.7 (Alpha variant), B.1.351 (Beta variant), B.1.526 (Iota variant), P.1 (Gamma variant) and B.1.617.2 (Delta variant). Consistent with previousreports^13,15–17^, neutralizing activity against the variants was lower than that against the original Wuhan-Hu-1 strain (Fig. 1e and Supplementary Table 3). Initial geometric mean neutralizing titres at 1.3 months against B.1.351, B.1.1.7, B.1.526, P.1 and B.1.617.2 were 5.7-, 1.8-, 1.1-, 1.4- and 2.7-fold lower, respectively, than they were against the Wuhan-Hu-1 virus (Fig. 1e). In the months following vaccination, there was a decrease in neutralizing activity against Wuhan-Hu-1 (R683G) and all the variants, with geometric mean neutralizing titres for wild-type (WT), B.1.351, B.1.1.7, B.1.526, P.1 and B.1.617.2 strains decreasing by 2.9-, 1.8-, 2.3-, 2.9-, 2.4- and 2.6-fold, respectively (Fig. 1e and Supplementary Table 3).

## Monoclonal antibodies

**Fig. 2:**
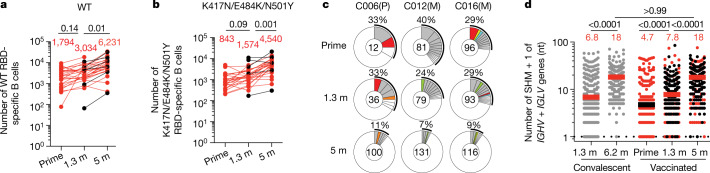
Anti-SARS-CoV-2 RBD B cells after vaccination. **a**, **b**, Graphs summarizing the number of Wuhan-Hu-1 RBD (WT)-specific memory B cells (**a**) and the number of antigen-specific memory B cells cross-reactive with both WT and K417N/E484K/N501Y mutant RBD (**b**) per 10 million B cells for *n* = 32 individuals after prime and 1.3 and 5 months after full vaccination. Samples without a prime value are shown in black. **c**, Pie charts showing the distribution of IgG antibody sequences obtained for memory B cells from three representative individuals after prime and 1.3 and 5 months after the boost. Additional pie charts can be found in Extended Data Fig. 3. The number inside the circle indicates the number of sequences analysed for the individual denoted above the circle, with Pfizer-BioNTech vaccine indicated by (P) and Moderna vaccine indicated by (M). Pie slice size is proportional to the number of clonally related sequences. The black outline and associated numbers indicate the percentage of clonally expanded sequences detected at each time point. Coloured slices indicate persisting clones (same *IGHV* and *IGLV* genes, with highly similar complementarity-determining region 3 sequences (CDR3s)) found at more than one time point within the same individual, grey slices indicate clones unique to the time point and white slices indicate repeating sequences isolated only once per time point. **d**, Number of nucleotide (nt) somatic hypermutations (SHM) in *IGHV* and *IGLV* genes combined (*n* = 2,050; Supplementary Table 4) in the antibodies illustrated in **c** and Extended Data Fig. 3, compared with the number of mutations obtained 1.3 months (ref. ^3^) and 6.2 months (ref. ^7^) after infection (grey). Horizontal bars and red numbers indicate the mean value at each time point. Samples without a prime value are shown in black. Statistical significance in **a**, **b** and **d** was determined by two-tailed Kruskal–Wallis test with subsequent Dunn’s multiple-comparisons test.

**Extended Data Fig. 2:**
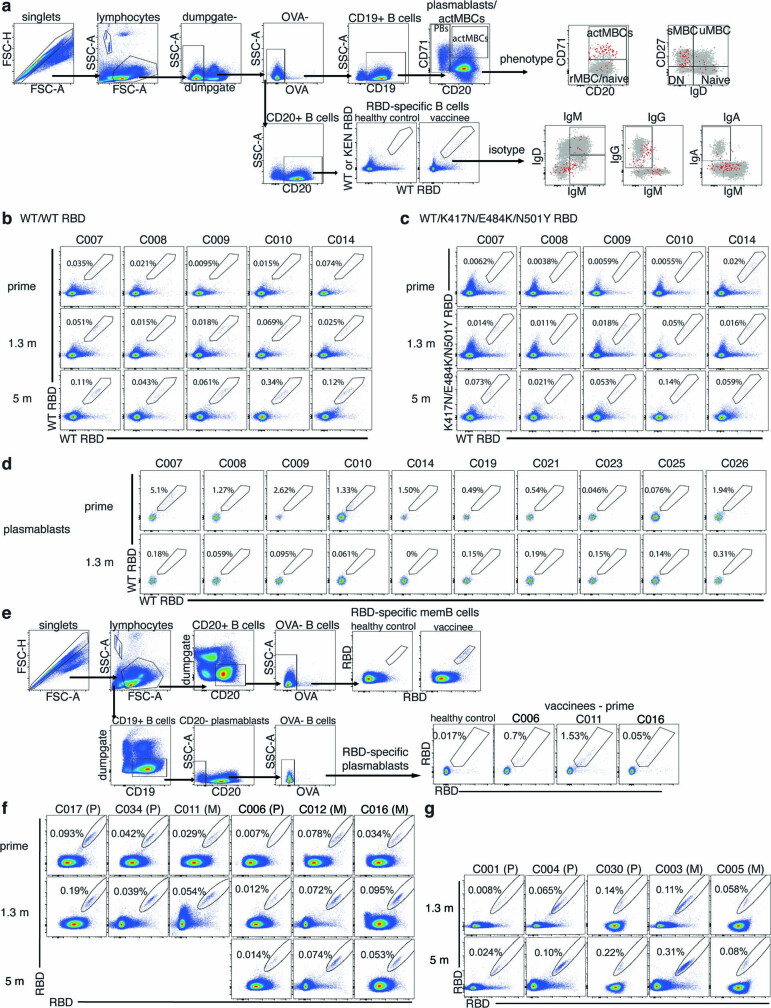
Flow Cytometry. **a**, Gating strategy for phenotyping. Gating was on singlets that were CD19^+^ or CD20^+^ and CD3-CD8-CD16-Ova-. Anti-IgG, IgM, IgA, IgD, CD71 and CD27 antibodies were used for B cell phenotype analysis. Antigen-specific cells were detected based on binding to RBD WT-PE^+^ and RBD WT/KEN (K417N/E484K/N501Y)-AF647^+^. **b-c**, Flow cytometry plots showing the frequency of **b**, RBD WT-binding memory B cells, and **c**, RBD-binding memory B cells cross-reactive with WT and K417N/E484K/N501Y mutant RBD in 5 selected individuals, after prime, 1.3 months, and 5 months post-second vaccination. **d**, Flow cytometry plots showing frequency of RBD-binding plasmablasts, in 10 selected vaccinees after prime or 1.3 months post-boost. **e**, Gating strategy for single-cell sorting for CD20+ memory B cells (top panel) or CD19+CD20- plasmablasts (bottom panel) which were double positive for RBD-PE and RBD-AF647. **f-g**, Representative flow cytometry plots showing dual AlexaFluor-647-RBD and PE-RBD-binding, single-cell sorted B cells from **f**, 6 individuals after prime and 1.3 months or 5 months post-boost and **g**, 5 individuals from 1.3- or 5-months post-boost. Percentage of RBD-specific B cells is indicated.

**Extended Data Fig. 3:**
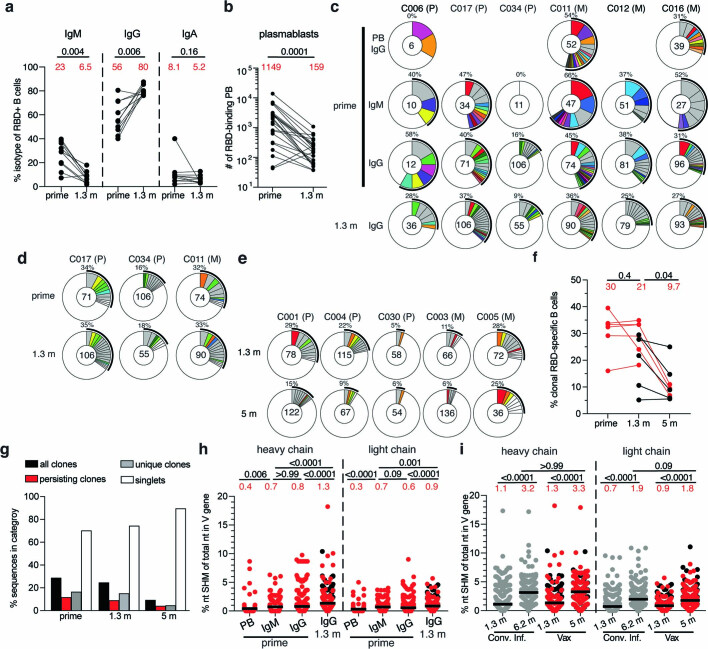
anti-SARS-CoV-2 RBD-specific plasmablast and memory B cells responses after vaccination. **a-b**, Graph showing the **a**, frequency of IgM, IgG, or IgA isotype expression by Wuhan-Hu RBD-specific memory B cells after prime or 1.3 months post-boost (n=10), and **b**, number of Wuhan-Hu RBD-binding plasmablasts per 10 million B cells (n=26) after prime or 1.3 months post-boost. Red numbers indicate geometric means. Gating strategy is in Extended Data Fig. 2. **c-e**, Pie charts show the distribution of IgG antibody sequences obtained from **c**, 6 individuals after prime (upper panel) or 1.3 months post-boost (lower panel). Sequences derived from IgG plasmablast (PB), IgM memory B cells (MBC), and IgG MBC compartments were analyzed after prime, while only IgG MBCs were analyzed at 1.3 months after boost, as indicated to the left of the plots. Pie charts showing only IgG memory B cells from 8 individuals (in additional to the 3 vaccinees shown in Fig. 2c) after **d**, prime and 1.3-months post-boost and **e**, 1.3- and 5-months post-boost. The number inside the circle indicates the number of sequences analyzed for the individual denoted above the circle, with Pfizer vaccinees indicated by (P) and Moderna by (M). Pie slice size is proportional to the number of clonally related sequences. The black outline and associated numbers indicate the percentage of clonally expanded sequences detected at each time point. Colored slices indicate persisting clones (same *IGHV* and *IGLV* genes, with highly similar CDR3s) found at more than one timepoint within the same individual. Grey slices indicate clones unique to the timepoint. White slices indicate repeating sequences isolated only once per time point. **f**, Graph shows the relative percentage of clonal sequences of IgG memory B cells at each time point from n=11 vaccinated inviduals illustrated in Fig. 2c and Extended Data Fig. 3d, e. The red numbers indicate the geometric means. Samples without a prime value are shown in black. **g**, Graph shows the percentage of total paired-sequences from IgG memory B cells (n=2050) analyzed at either prime, 1.3- or 5-months post-boost, that can be found as part of all clones (black bars), persisting clones (red bars), unique clones (grey bars), or singlets (white bar). **h-i**, Ratio of the number of somatic nucleotide mutations over the nucleotide length of the V gene in the Ig heavy and light chains, separately, in antibodies detected in **h**, different B cell compartments after prime or 1.3 months post-boost (n=1565) and **i**, IgG memory B cells at 1.3 or 5 months post-boost (n=1610) compared to convalescent infected (grey) individuals after 1.3^3^ and 6.2^7^ months post-infection (also Supplementary Table 4). Horizontal bars and red numbers indicate mean ratio in each compartment at each time point. Sequences derived from samples without a prime value are shown in black. Statistical significance in **a** and **b** was determined using a two-tailed Wilcoxon matched-pairs signed rank test. **f, h**, and **i** was determined by two-tailed Kruskal Wallis test with subsequent Dunn’s multiple comparisons.

Circulating antibodies produced by plasma cells can prevent infection if present at sufficiently high concentrations at the time of exposure. By contrast, the memory B cell compartment contains long-lived antigen-specific B cells that mediate rapid recall responses that contribute to long-term protection^18^. To examine the nature of the memory compartment elicited by one or two mRNA vaccine doses and its evolution after 5 months, we used flow cytometry to enumerate B cells expressing receptors that bind to Wuhan-Hu-1 (WT) and B.1.351 (K417N/E484K/N501Y) RBDs (Fig. 2a, b, and Extended Data Fig. 2). Although neutralizing antibodies develop to other parts of the spike protein, we focused on the RBD because it is the dominant target of the memory antibody neutralizing response^19,20^. Wuhan-Hu-1 RBD-specific memory B cells developed after the prime in all volunteers examined, and their numbers increased for up to 5 months after vaccination (Fig. 2a). Memory B cells binding to the B.1.351 (K417N/E484K/N501Y) variant RBD were detectable but in lower numbers than B cells binding WT RBD in all samples examined (Fig. 2b). Whereas IgG-expressing memory cells increased in number after the boost, IgM-expressing memory B cells that made up 23% of the memory compartment after the prime were nearly absent after boosting (Extended Data Fig. 3a). Finally, circulating RBD-specific plasmablasts were readily detected after the prime but were infrequent after the boost (Extended Data Figs. 2d and 3b).

**Extended Data Fig. 4:**
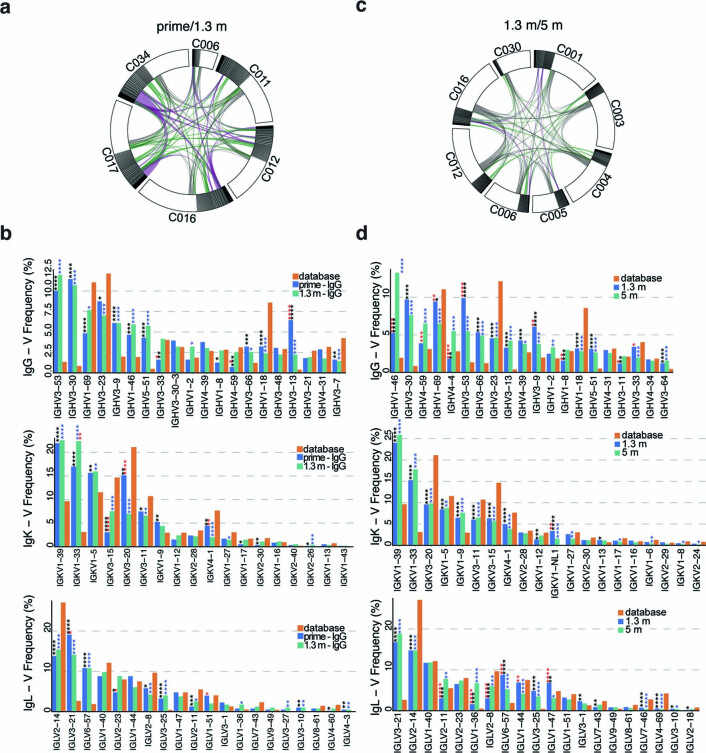
Frequency distribution of human V genes. **a**, Circos plot depicting relationship between antibodies that share V and J gene usage in both IgH and IgL when comparing prime/1.3m IgG MBC sequences. Purple, green, and grey lines connect related clones, clones and singlets, and singlets to each other, respectively. **b**, Graph shows relative abundance of human heavy chain *IGHV* (top), light chain *IGKV* (middle) or *IGLV* (bottom) genes comparing Sequence Read Archive accession SRP010970 (orange), and IgG MBCs after prime (blue) or 1.3 months post-boost (green). Statistical significance was determined by two-sided binomial test. * = p≤0.05, ** = p≤0.01, *** = p≤0.001, **** = p≤0.0001. Color of stars indicates: black - comparing Database versus Prime; blue - comparing Database versus 1.3m; red - comparing Prime versus 1.3m. **c**, Circos plot depicting relationship between antibodies that share V and J gene usage in both IgH and IgL when comparing 1.3 m/5 m IgG MBC sequences. Purple, green, and grey lines connect related clones, clones and singlets, and singlets to each other, respectively. **d**, Graph shows relative abundance of human heavy chain *IGHV* (top), light chain *IGKV* (middle) or *IGLV* (bottom) genes comparing Sequence Read Archive accession SRP010970 (orange), and IgG MBCs after 1.3 months (blue) or 5 months (green) post-vaccination. Statistical significance was determined by two-sided binomial test. * = p≤0.05, ** = p≤0.01, *** = p≤0.001, **** = p≤0.0001. Color of stars indicates: black - comparing Database versus 1.3 months; blue - comparing Database versus 5 months; red - comparing 1.3 months versus 5 months.

The memory compartment continues to evolve up to 1 year after natural infection, with selective enrichment of cells producing broad and potent neutralizing antibodies^1^. To determine how the memory compartment evolves after vaccination, we obtained 2,327 paired antibody sequences from 11 individuals sampled at the time points described above (Fig. 2c, Extended Data Fig. 3c–e and Supplementary Table 4). As expected, *IGHV3-30* and *IGHV3-53* were over-represented after the first and second vaccine dose and remained over-represented 5 months after vaccination^21–23^ (Extended Data Fig. 4).

**Extended Data Fig. 5:**
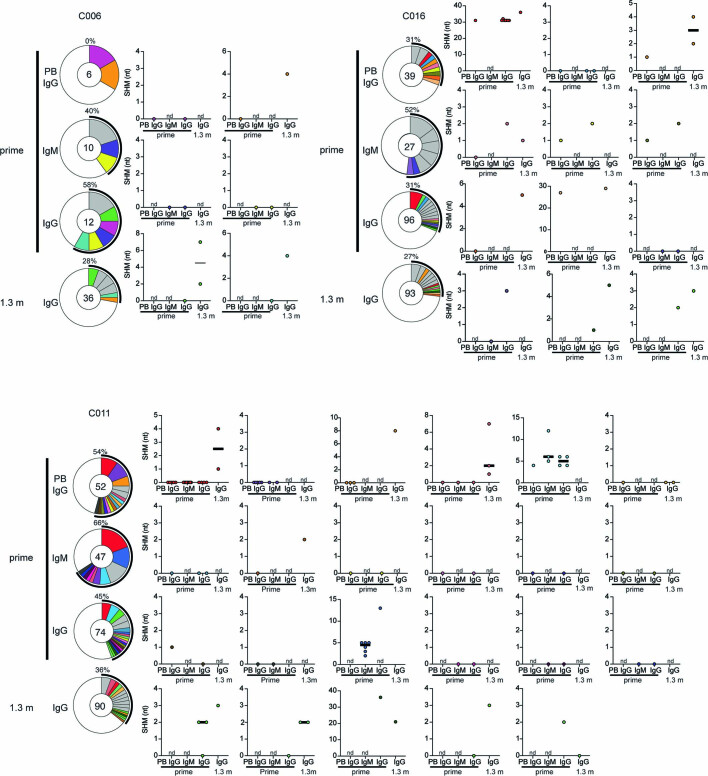
Somatic hypermutation of anti-SARS-CoV-2 RBD antibody clones after prime or boost. Clonal evolution of RBD-binding B cells from 3 individuals for which plasmablasts, IgM memory B cells, and IgG memory B cells were analyzed after prime, and IgG memory B cells were analyzed after 1.3 months post-boost (as described in Extended Data Fig. 3). The number of somatic nucleotide mutations found in shared clonal families found in at least 2 different compartments is graphed to the right of each donut plot. Color of dot plots match the color of pie slices within the donut plot, which indicate persisting clones. nd – clone was Not Detected in the indicated compartment. Black horizontal line indicates median number of SHM.

All individuals examined showed expanded clones of memory B cells that expressed closely related *IGHV* and *IGHL* genes (Fig. 2c and Extended Data Figs. 3c–e and 4). Paired samples from prime and 1.3 months after the boost showed expanded clones of memory B cells, some of which were shared across plasmablasts, IgM- and IgG-expressing cells at prime, and IgG-expressing memory cells after boost (Extended Data Figs. 3c and 5). Thus, the cell fate decision controlling germinal centre versus plasmablast cell fate is not entirely affinity dependent, as cells with the same initial affinity can enter both compartments to produce clonal relatives^24^.

The relative fraction of memory cells found in expanded clones varied between prime and boost and among individuals and decreased over time (Fig. 2c and Extended Data Fig. 3d–f). Overall, these clones represented 30%, 21% and 9.7% of all sequences after prime and at the 1.3- and 5-month time points, respectively (Extended Data Fig. 3f). Nevertheless, clones of memory B cells continued to evolve for up to 5 months in vaccinated individuals, as evidenced by the appearance of unique clones. Notably, unique clones appearing after 1.3 and 5 months represented a greater or equal fraction of the total memory B cell pool relative to persisting clones (16% versus 9.6% and 5.1% versus 4.7%, respectively) (Fig. 2c and Extended Data Fig. 3d, e, g). Finally, memory B cells emerging after the boost showed significantly higher levels of somatic mutations than plasmablasts or memory B cells isolated after the prime, and they continued to accumulate mutations up to 5 months after the boost (Fig. 2d and Extended Data Fig. 3h, i). In conclusion, the memory B cell compartment continues to evolve for up to 5 months after mRNA vaccination.

## Neutralizing activity of monoclonal antibodies

**Extended Data Fig. 6:**
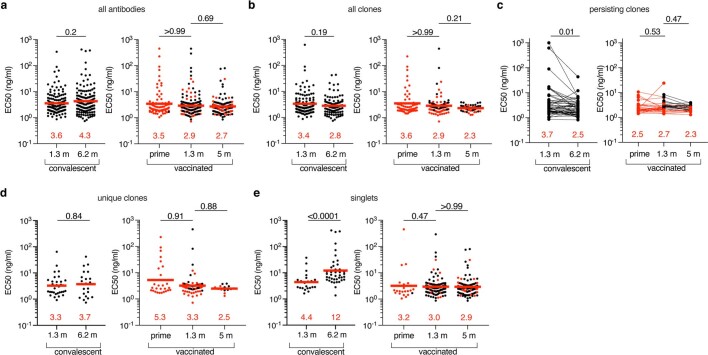
Anti-SARS-CoV-2 RBD monoclonal antibodies ELISAs. **a-e**, Graphs show anti-SARS-CoV-2 binding activity of n=458 monoclonal antibodies measured by ELISA against RBD. ELISA half-maximal concentration (EC_50_) values for all antibodies (**a**), all clones (**b**), persisting clones (**c**), unique clones (**d**) and singlets (**e**) isolated from COVID-19 convalescent individuals 1.3^3^ and 6.2^7^ months after infection (left panel) or from vaccinated individuals after prime, or 1.3m or 5m after receiving the second dose of mRNA vaccination (right panel). Each dot represents one antibody. Antibodies isolated from samples without a prime value are shown in black. Red horizontal bars and numbers indicate geometric mean values. Statistical significance was determined by two-tailed Mann-Whitney test (left panels of **a, b, d and e**), two-tailed Kruskal-Wallis test with subsequent Dunn’s multiple comparisons (right panels of **a-e**) or by two-tailed Wilcoxon test (left panel of **c**). All experiments were performed at least twice.

We performed ELISAs to confirm that the antibodies isolated from memory B cells bind to the RBD (Extended Data Fig. 6). In total, 458 antibodies were tested by ELISA, including 88 isolated after the first vaccine dose, 210 isolated after the boost and 160 isolated from individuals who had been fully vaccinated 5 months earlier. Among the 458 antibodies tested, 430 (94%) bound to the Wuhan-Hu-1 RBD, indicating that the method used to isolate RBD-specific memory B cells was highly efficient (Supplementary Tables 5–6). The geometric mean ELISA half-maximal effective concentration (EC_50_) of the antibodies obtained after prime and 1.3 and 5 months after the second dose was 3.5, 2.9 and 2.7 ng ml^–1^, respectively, suggesting no major change in binding over time after vaccination (Extended Data Fig. 6 and Supplementary Tables 5, 6).

**Fig. 3:**
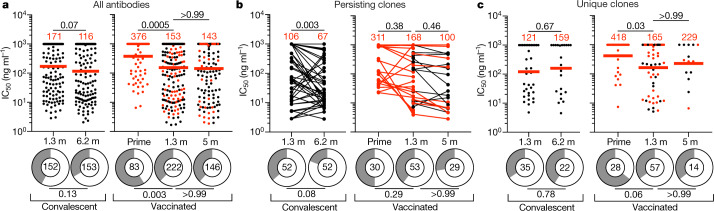
Anti-SARS-CoV-2 RBD monoclonal antibodies. **a**–**c**, Graphs showing the anti-SARS-CoV-2 neutralizing activity of monoclonal antibodies measured by SARS-CoV-2-pseudotyped virus neutralization assays using WT (Wuhan-Hu-1; ref. ^50^) SARS-CoV-2 pseudovirus^3,8^. IC_50_ values for all antibodies (**a**), persisting clones (**b**) and unique clones (**c**) isolated from convalescent individuals 1.3 months (ref. ^3^) and 6.2 months (ref. ^7^) after infection or from vaccinated individuals after prime and 1.3 and 5 months after the boost are shown. Each dot represents one antibody; 451 total antibodies were tested, including the 430 reported herein (Supplementary Table 5) and 21 previously reported antibodies^13^. Antibodies isolated from samples without a prime value are shown in black. Pie charts illustrate the fraction of non-neutralizing (IC_50_ > 1,000 ng ml^–1^) antibodies (grey slices); the inner circle shows the number of antibodies tested per group. Horizontal bars and red numbers indicate geometric mean values. Statistical significance was determined by two-tailed Kruskal–Wallis test with subsequent Dunn’s multiple-comparisons test and for ring plots was determined by two-tailed Fisher’s exact test with subsequent Bonferroni correction. All experiments were performed at least twice.

**Extended Data Fig. 7:**
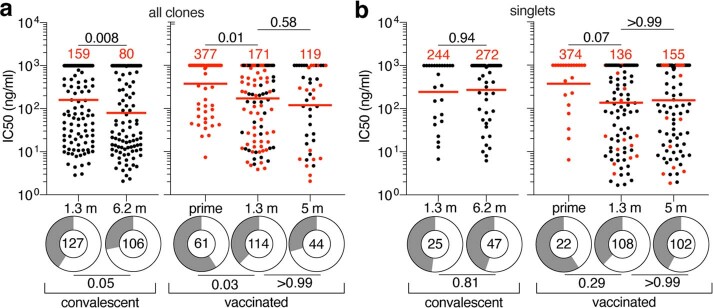
Anti-SARS-CoV-2 RBD monoclonal antibodies. **a-c**, Graphs show anti-SARS-CoV-2 neutralizing activity of monoclonal antibodies measured by a SARS-CoV-2 pseudotype virus neutralization assay using wild-type (Wuhan Hu-1^50^) SARS-CoV-2 pseudovirus^3,8^. Half-maximal inhibitory concentration (IC_50_) values for antibodies from **a**, all clones and **e**, singlets isolated from COVID-19 convalescent individuals 1.3^3^ and 6.2^7^ months after infection or from vaccinated individuals after prime, and 1.3- or 5-months after 2 doses of vaccine. Each dot represents one antibody, where 451 total antibodies were tested including the 430 reported herein (Supplementary Table 5), and 21 previously reported antibodies^13^. Antibodies isolated from samples without a prime value are shown in black. Pie charts illustrate the fraction of non-neutralizing (IC50 > 1000 ng/ml) antibodies (grey slices), inner circle shows the number of antibodies tested per group. Horizontal bars and red numbers indicate geometric mean values. Statistical significance was determined by two-tailed Kruskal Wallis test with subsequent Dunn’s multiple comparisons, and for ring plots by two-tailed Fisher’s exact test with subsequent Bonferroni-correction. All experiments were performed at least twice.

In total, 430 RBD-binding antibodies were tested for neutralizing activity using HIV-1 pseudotyped with the SARS-CoV-2 spike protein^3,8^. The geometric mean half-maximal inhibitory concentration (IC_50_) of RBD-specific memory antibodies improved from 376 ng ml^–1^ to 153 ng ml^–1^ between the first and second vaccine dose (*P* = 0.0005) (Fig. 3a). The improvement was reflected in all clones (IC_50_ = 377 versus 171 ng ml^–1^, *P* = 0.01) (Extended Data Fig. 7a), persisting clones (IC_50_ = 311 versus 168 ng ml^–1^) (Fig. 3b and Supplementary Table 6), unique clones (IC_50_ = 418 versus 165 ng ml^–1^, *P* = 0.03) (Fig. 3c) and single antibodies (IC_50_ = 374 versus 136 ng ml^–1^) (Extended Data Fig. 7b). The increase in neutralizing activity between the first and second vaccine doses was associated with a decrease in the percentage of non-neutralizing antibodies (defined as having IC_50_ >1,000 ng ml^–1^) and increased representation of neutralizing antibodies (*P* = 0.003) (Fig. 3a). In conclusion, memory B cells recruited after the second dose account for most of the improvement in neutralizing activity in this compartment when comparing the two vaccine doses. Thus, in addition to the quantitative improvement in serum neutralizing activity, there is also an improvement in the neutralizing activity of the antibodies expressed in the memory compartment after boosting.

By contrast, there was no significant improvement in neutralizing activity when comparing the monoclonal antibodies obtained 5 months after vaccination with those obtained at 1.3 months (*P* > 0.99) (Fig. 3a). Although there was some improvement among B cell clones, which was accounted for by the small minority of persisting clones, this was not significant in either group (*P* = 0.58 and 0.46) (Fig. 3b, Extended Data Fig. 7a and Supplementary Table 6). By contrast, memory antibodies obtained from convalescent individuals showed improved neutralizing activity at 6.2 months (ref. ^7^) relative to 1.3 months (ref. ^3^), with a decrease in IC_50_ from 171 ng ml^–1^ to 116 ng ml^–1^ (Fig. 3a), and neutralizing activity was further improved after 1 year^1^. This improvement was due to increased neutralizing activity among persisting clones (*P* = 0.003) (Fig. 3b).

## Affinity, epitopes and neutralization breadth

**Fig. 4:**
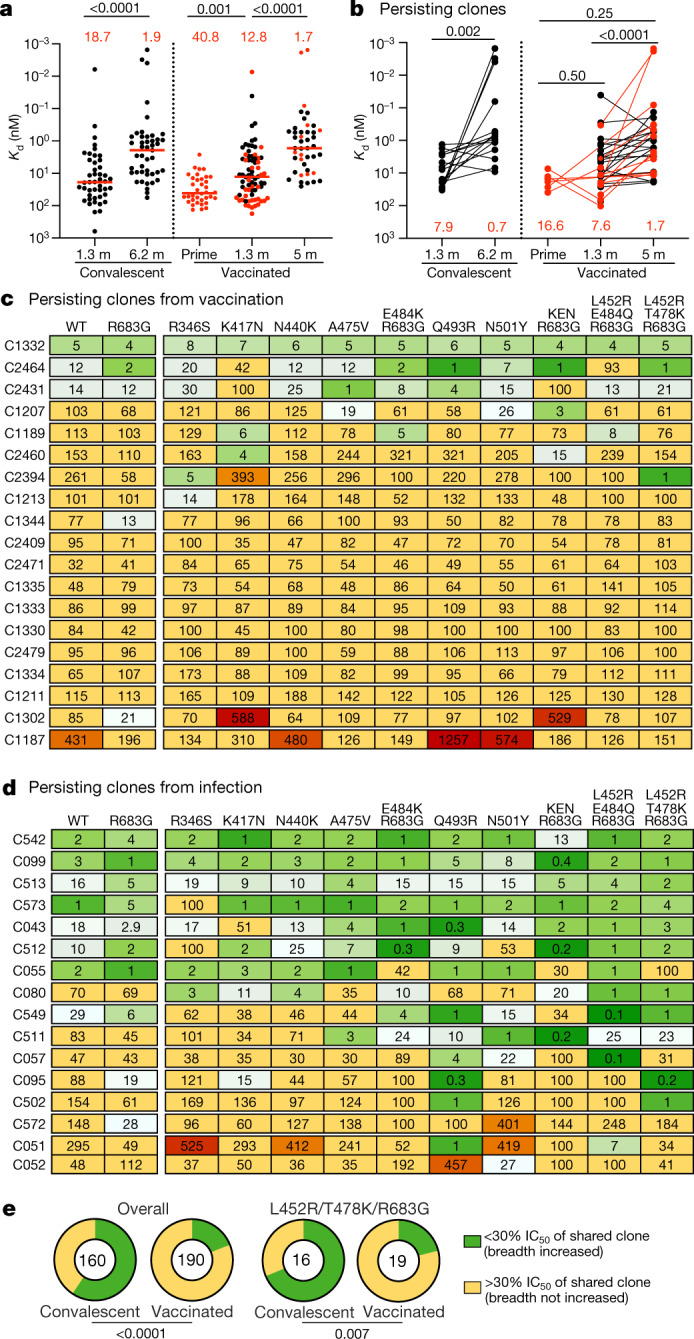
Affinity and breadth. **a**, **b**, Graphs showing antibody dissociation constant (*K*_d_) values for Wuhan-Hu-1 RBD measured by BLI. **a**, Antibodies isolated from convalescent individuals 1.3 months (*n* = 42)^3^ and 6.2 months (*n* = 45)^7^ after infection or from vaccinated individuals after prime (*n* = 36) and 1.3 months (*n* = 74) and 5 months (*n* = 43) after the second vaccination. **b**, Clonally paired antibodies isolated from convalescent individuals 1.3 months (ref. ^3^) and 6.2 months (ref. ^7^) after infection (*n* = 15) or vaccinated individuals at prime and 1.3 months (*n* = 3), at prime and 5 months (*n* = 3), or at 1.3 and 5 months after full vaccination (*n* = 26). Antibodies isolated from samples without a prime value are shown in black. Red horizontal bars and numbers indicate median values. **c**, **d**, Heat maps showing inhibitory concentrations of antibodies isolated 5 months after vaccination (**c**) or 6.2 months (ref. ^7^) after infection (**d**) normalized to their shared clone isolated 1.3 months after vaccination (**c**) or 1.3 months (ref. ^3^) after infection (**d**), expressed as %IC_50_, against the indicated WT or mutant SARS-CoV-2 pseudoviruses (Supplementary Table 8). Antibodies with improved (<30%) IC_50_ compared with their clonal relative isolated at an earlier time point are shown in shades of green with the most improved antibodies in dark green. Antibodies with worse (>300%) IC_50_ than their clonal relative isolated at an earlier time point are shown in red with the most worsened antibodies in dark red. Antibodies for which IC_50_ did not change by more than around 3-fold are shown in yellow. **e**, Pie charts illustrating the fraction of antibodies showing improved (<30%, green) versus not improved (yellow) IC_50_ values compared with their clonal relative isolated at an earlier time point. The inner circle shows the number of antibody–mutant combinations analysed per group. Statistical significance in **a** and **b** was determined using two-tailed Kruskal–Wallis test with subsequent Dunn’s multiple-comparisons test and in **e** was determined by two-tailed Fisher’s exact test with subsequent Bonferroni correction.

**Extended Data Fig. 8:**
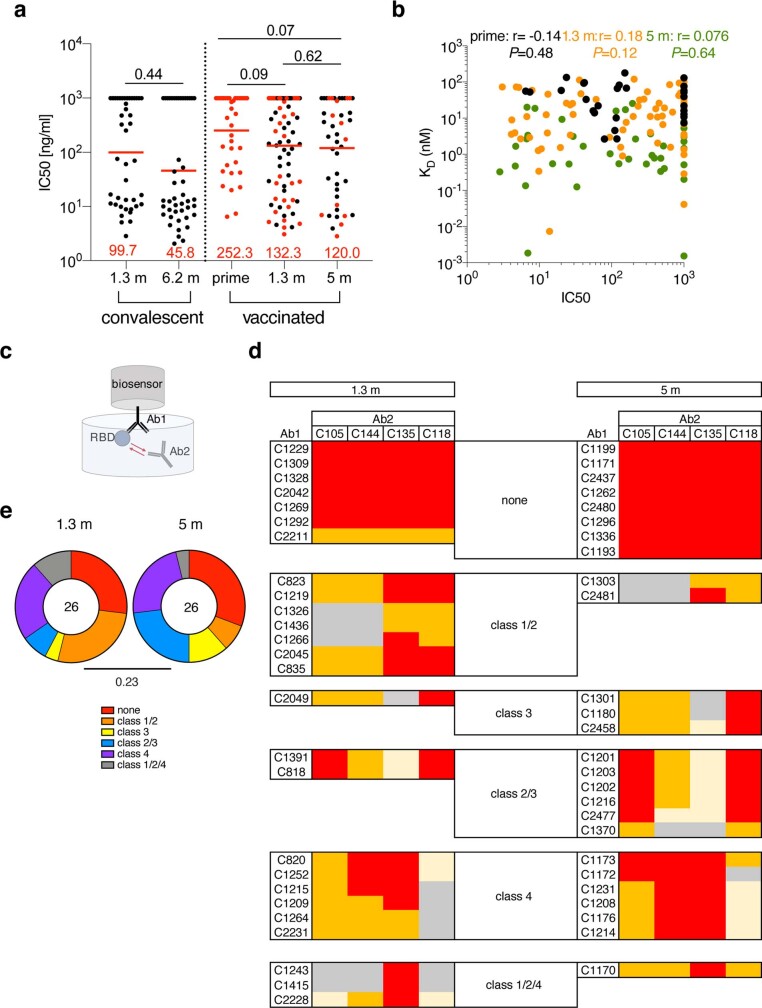
Affinity and Epitope targeting of anti-SARS-CoV-2 RBD antibodies. **a**, IC_50_ values for randomly selected antibodies isolated from convalescents 1.3m^3^ (n=42) and 6.2m^7^ (n=45) after infection or from vaccinees after prime (n=36), and 1.3m (n=74) and 5m (n=43). Red horizontal lines and numbers indicate geometric mean. Antibodies isolated from samples without a prime value are shown in black. **b**, Graphs show affinities (K_D_, Y-axis) plotted against neutralization activity (IC_50_, X-axis) for antibodies isolated after prime (black), or 1.3m (orange) or 5m (green) post-boost vaccination for antibodies showin in **a**. **c**, Schematic representation of the BLI experiment for randomly selected antibodies isolated from vaccinees 1.3- and 5 months after full vaccination (each presented group shows n=26 antibodies). **d**. Heat-map of relative inhibition of Ab2 binding to the preformed Ab1-RBD complexes (grey=no binding, yellow=low binding, orange=intermediate binding, red=high binding). Values are normalized through the subtraction of the autologous antibody control. BLI traces can be found in Extended Data Fig. 9. **e**. Pie charts indicate the fraction of antibodies that are assigned to different classes according to their binding pattern as shown in **d** and Extended data Fig. 9. Number in inner circle shows number of antibodies tested. Statistical significance was determined using a two-tailed Kruskal Wallis test with subsequent Dunn’s multiple comparisons in **a** and two-tailed Spearman correlation test in **b**, and a two-tailed Chi-square test in **e**.

To examine affinity maturation after vaccination, we performed biolayer interferometry (BLI) experiments using the Wuhan-Hu-1 RBD^3^. In total, 147 antibodies were assayed, 30 obtained after the prime, 74 obtained 1.3 months after boosting and 43 obtained 5 months after boosting. Geometric mean IC_50_ values were comparable for the antibodies obtained from the 1.3- and 5-month time points (Extended Data Fig. 8a). Overall, there was a 3- and 7.5-fold increase in affinity for the antibodies obtained between the first two and between the second two time points, respectively (Fig. 4a). After 5 months, the affinity of the antibodies obtained from vaccinated individuals was similar to that for antibodies obtained from naturally infected volunteers (Fig. 4a). However, there was no correlation between the affinity and neutralizing activity of the antibodies tested at any of the three time points (Extended Data Fig. 8b).

We also compared the affinity for pairs of antibodies obtained from persisting clones at 1.3 and 5 months after vaccination. Persisting clones obtained at 5 months from vaccinated individuals showed a median 4.5-fold increase in affinity relative to the 1.3-month time point (*P* < 0.0001) (Fig. 4b). By contrast, a comparable group of persistingclonal antibodies obtained from convalescent individuals 1.3 and 6.2 months after infection showed a median 11.2-fold increase in affinity at the later time point (*P* = 0.002; Fig. 4b).

**Extended Data Fig. 9:**
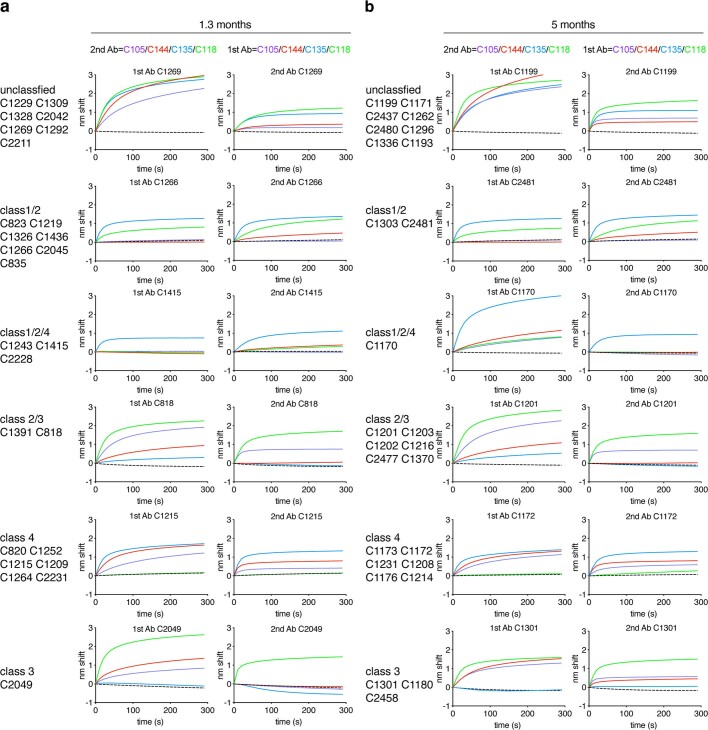
BLI traces from epitope mapping of anti-SARS-CoV-2 RBD antibodies. **a, b**, BLI traces from competition experiments used to determine epitope targets of anti-SARS-CoV-2 RBD antibodies isolated from vaccinees at 1.3m (**a**) or 5m (**b**) post-boost, as illustrated in Extended Data Fig. 8.

To determine whether the epitopes targeted by the monoclonal antibodies were changing over time, we performed BLI experiments in which a preformed antibody–RBD complex was exposed to a second monoclonal antibody targeting one of four classes of structurally defined epitopes^1,3^ (see schematic in Extended Data Fig. 8c). There was no significant change in the distribution of targeted epitopes among 52 randomly selected antibodies, with comparable neutralizing activity obtained at the 1.3- and 5-month time points (Extended Data Figs. 8d, e and 9).

In addition to the increase in potency, the neutralizing breath of memory antibodies obtained from persisting clones in convalescent individuals increases with time after infection^1,7,25^. To determine whether there is a similar increase in breadth with time after vaccination, we randomlyselected 20 antibodies from prime or 1.3 months after boost with representative levels of activity against the original Wuhan-Hu-1 strain and measured their neutralization potency against a panel of pseudotyped viruses encoding RBD mutations that were selected for resistance to different anti-RBD antibody classes and/or are associated with circulating variants of concern (Extended Data Table 1). There was little change in breadth between prime and 1.3 months after boost, with only a small increase in resistance to variants with the K417N and A475V substitutions (Extended Data Table 1 and Supplementary Table 7).

In addition, we assayed 19 pairs of neutralizing antibodies expressed by persisting clones obtained 1.3 and 5 months after vaccination for their potency against the same mutant pseudotype viruses (Fig. 4c and Supplementary Table 8). These were compared to seven previously reported^25^ and nine additional pairs of antibodies obtained from convalescent individuals at 1.3- and 6.2-month time points (Fig. 4d and Supplementary Table 8). Whereas only 36 of 190 (19%) of the antibody–mutant combinations in vaccinated individuals showed improved potency at the later time point, 95 of the 160 (59%) pairs in convalescent individuals exhibited an increase in potency (*P* < 0.0001) (Fig. 4c–e). Moreover, only 4 of the 19 (21%) antibody pairs from vaccinated individuals showed improved potency against pseudotypes carrying B.1.617.2 (Delta variant)-specific RBD amino acid substitutions (L452R/T478K), while 11 of 16 (69%) of the convalescent antibody pairs showed improved activity against this virus (*P* = 0.007) (Fig. 4c–e). We conclude that there is less increase in breadth in the months after mRNA vaccination than there is in a similar interval in naturally infected individuals.

Circulating antibodies are produced by an initial burst of short-lived plasmablasts^26,27^ and maintained by plasma cells with variable longevity^28,29^. SARS-CoV-2 infection or mRNA vaccination produces an early peak antibody response that decreases by 5- to 10-fold after 5 months^7,30–34^. Notably, neutralization titres elicited by vaccination exceed those in individuals who have recovered from COVID-19 at all comparable time points assayed. Nevertheless, neutralizing potency against variants is significantly lower than against Wuhan-Hu-1, with up to 5- to 10-fold-reduced activity against the B.1.351 variant^5,6,13,14,35^. Taken together with the overall decay in neutralizing activity, there can be a decrease of 1–2 orders of magnitude in serum neutralizing activity against variants after 5 or 6 months when compared with the peak neutralizing activity against Wuhan-Hu-1. Thus, antibody-mediated protection against variants is expected to wane significantly over a period of months, consistent with reports of re-infection in convalescent individuals and breakthrough infection by variants in fully vaccinated individuals^36–39^.

In contrast to circulating antibodies, memory B cells are responsible for rapid recall responses^40–42^, and the number of cells in this compartment is relatively stable over the first 5–6 months after mRNA vaccination or natural infection^7,43^. In both cases, memory B cells continue to evolve, as evidenced by increasing levels of somatic mutation and emergence of unique clones.

The memory response would be expected to protect individuals who experience breakthrough infection from developing serious disease. Both natural infection and mRNA vaccination produce memory antibodies that evolve increased affinity. However, vaccine-elicited memory monoclonal antibodies show more modest neutralizing potency and breadth than those that develop after natural infection^1,7^. Notably, the difference between the memory compartments that develop in response to natural infection versus mRNA vaccination reported above is consistent with the higher level of protection from variants conferred by natural infection^39^.

There are innumerable differences between natural infection and mRNA vaccination that could account for the differences in antibody evolution over time. These include, but are not limited to, (1) route of antigen delivery (respiratory tract versus intramuscular injection)^44,45^; (2) the physical nature of the antigen (intact virus versus conformationally stabilized pre-fusion spike protein)^46^; and (3) antigen persistence (weeks in the case of natural infection^7^ versus hours to days for mRNA vaccination)^47^. Each of these factors could affect B cell evolution and selection directly and indirectly through differential T cell recruitment.

The increase in potency and breadth in the memory compartment that develops after natural infection accounts for the exceptional responses to Wuhan-Hu-1 and its variants that convalescent individuals exhibit when boosted with mRNA vaccines^1,5^. The expanded memory B cell compartment in individuals receiving mRNA vaccines should also produce high titres of neutralizing antibodies when these individuals receive boosts or when they are re-exposed to the virus^48^. Boosting vaccinated individuals with currently available mRNA vaccines should produce strong responses that mirror or exceed the initial vaccine responses to Wuhan-Hu-1, but with similarly decreased coverage against variants. Whether an additional boost with Wuhan-Hu-1-based or variant vaccines or re-infection will also elicit development of memory B cells expressing antibodies showing increased breadth remains to be determined. Finally, timing an additional boost for optimal responses depends on whether the objective is to prevent infection or disease^49^. Given the current rapid emergence of SARS-CoV-2 variants, boosting to prevent infection would probably be needed on a timescale of months. The optimal timing for boosting to prevent serious disease will depend on the stability and further evolution of the memory B cell compartment.

## Methods

### Study participants

Participants were healthy volunteers receiving either the Moderna (mRNA-1273) or Pfizer-BioNTech (BNT162b2) mRNA vaccine against SARS-CoV-2 who were recruited for serial blood donations at Rockefeller University Hospital in New York between 21 January and 20 July 2021. The majority of participants (*n* = 28) were de novo recruited for this study, while a subgroup of individuals (*n* = 4) were from a long-term study cohort^13^. Eligible participants were healthy adults with no history of infection with SARS-CoV-2, as determined by clinical history and confirmed through serology testing, receiving one of the two Moderna (mRNA-1273) or Pfizer-BioNTech (BNT162b2) vaccines according to current dosing and interval guidelines. Exclusion criteria included incomplete vaccination status, presence of clinical signs and symptoms suggestive of acute infection with SARS-CoV-2, a positive RT–PCR result for SARS-CoV-2 in saliva or positive COVID-19 serology. Seronegativity for COVID-19 was established through the absence of serological activity towards the nucleocapsid (N) protein of SARS-CoV-2. Participants presented to the Rockefeller University Hospital for blood sample collection and were asked to provide details of their vaccination regimen, possible side effects, comorbidities and possible COVID-19 history. Clinical data collection and management were carried out using the software iRIS by iMedRIS (v.11.02). All participants provided written informed consent before participation in the study, and the study was conducted in accordance with Good Clinical Practice principles. The study was performed in compliance with all relevant ethical regulations, and the protocol (DRO-1006) for studies with human participants was approved by the institutional review board of The Rockefeller University. For detailed participant characteristics, see Supplementary Tables 1 and 2.

### Blood sample processing and storage

Peripheral blood mononuclear cells (PBMCs) obtained from samples collected at Rockefeller University were purified as previously reported by gradient centrifugation and stored in liquid nitrogen in the presence of foetal calf serum (FCS) and DMSO^3,7^. Heparinized plasma and serum samples were aliquotted and stored at –20 °C or below. Before experiments, aliquots of plasma samples were heat inactivated (56 °C for 1 h) and then stored at 4 °C.

### ELISAs

ELISAs^51,52^ to evaluate antibodies binding to SARS-CoV-2 RBD were performed by coating high-binding 96-half-well plates (Corning, 3690) with 50 μl per well of a 1 μg ml^–1^ protein solution in PBS overnight at 4 °C. Plates were washed six times with washing buffer (1× PBS with 0.05% Tween-20 (Sigma-Aldrich)) and incubated with 170 μl per well of blocking buffer (1× PBS with 2% BSA and 0.05% Tween-20 (Sigma)) for 1 h at room temperature. Immediately after blocking, monoclonal antibodies or plasma samples were added in PBS and plates wereincubated for 1 h at room temperature. Plasma samples were assayed at a 1:66 starting dilution with 10 additional threefold serial dilutions. Monoclonal antibodies were tested at a 10 μg ml^–1^ starting concentration with 10 additional fourfold serial dilutions. Plates were washed six times with washing buffer and then incubated with anti-human IgG, IgM or IgA secondary antibody conjugated to horseradish peroxidase (HRP) (Jackson Immuno Research, 109-036-088 and 109-035-129; Sigma, A0295) in blocking buffer at a 1:5,000 dilution (IgM and IgG) or a 1:3,000 dilution (IgA). Plates were developed by addition of the HRP substrate 3,3′,5,5′-tetramethylbenzidine (TMB) (ThermoFisher) for 10 min (plasma samples) or 4 min (monoclonal antibodies). The developing reaction was stopped by adding 50 μl of 1 M H_2_SO_4_, and absorbance was measured at 450 nm with an ELISA microplate reader (FluoStar Omega, BMG Labtech) with Omega and Omega MARS software for analysis. For plasma samples, a positive control (plasma from participant COV72, diluted 66.6-fold with 10 additional threefold serial dilutions in PBS) was added to every assay plate for normalization. The average of its signal was used for normalization of all other values on the same plate with Excel software before calculating the AUC using Prism v9.1(GraphPad). Negative controls of pre-pandemic plasma samples from healthy donors were used for validation (for more details, see ref. ^3^). For monoclonal antibodies, the ELISA EC_50_ was determined using four-parameter nonlinear regression (GraphPad Prism v9.1). EC_50_ values above 2,000 ng ml^–1^ were considered to correspond to non-binders.

### Proteins

The mammalian expression vector encoding the RBD of SARS-CoV-2 (GenBank MN985325.1; spike protein residues 319–539) was previously described^53^.

### SARS-CoV-2-pseudotyped reporter virus

The panel of plasmids expressing RBD-mutant SARS-CoV-2 spike proteins in the context of pSARS-CoV-2-S_Δ19_ has been described^13,25,54^. Variant pseudoviruses resembling variants of interest/concern B.1.1.7 (first isolated in the UK), B.1.351 (first isolated in South Africa), B.1.526 (first isolated in New York), P.1 (first isolated in Brazil) and B.1.617.2 (first isolated in India) were generated by introduction of substitutions using synthetic gene fragments (IDT) or overlap extension PCR-mediated mutagenesis and Gibson assembly. Specifically, the variant-specific deletions and substitutions introduced were as follows: B.1.1.7: ΔH69/V70, ΔY144, N501Y, A470D, D614G, P681H, T761I, S982A, D118H; B.1.351: D80A, D215G, L242H, R246I, K417N, E484K, N501Y, D614G, A701V; B.1.526: L5F, T95I, D253G, E484K, D614G, A701V; P.1: L18F, T20N, P26S, D138Y, R190S, K417T, E484K, N501Y, D614G, H655Y, T1027I, V1167F; B.1.617.2: T19R, Δ156–158, L452R, T478K, D614G, P681R, D950N.

The E484K, K417N/E484K/N501Y, L452R/E484Q and L452R/T478K substitutions, as well as the deletions/substitutions corresponding to the variants of concern listed above, were incorporated into a spike protein that also included the R683G substitution, which disrupts the furin cleavage site and increases particle infectivity. Neutralizing activity against mutant pseudoviruses was compared to that against a WT SARS-CoV-2 spike sequence (NC_045512), carrying R683G where appropriate.

SARS-CoV-2-pseudotyped particles were generated as previously described^3,8^. In brief, 293T (CRL-11268) and HT1080 (CCL-121) cells were obtained from ATCC. Cells were transfected with pNL4-3ΔEnv-nanoluc and pSARS-CoV-2-S_Δ19_ particles were collected 48 h after transfection, filtered and stored at –80 °C to propagate 293T/ACE2 and HT1080/ACE2.cl14 cells. Cell lines were checked for mycoplasma contamination by Hoeschst staining and confirmed negative.

### Pseudotyped virus neutralization assays

Fourfold serially diluted pre-pandemic negative-control plasma from healthy donors, plasma from COVID-19-convalescent individuals or monoclonal antibodies were incubated with SARS-CoV-2-pseudotyped virus for 1 h at 37 °C. The mixture was subsequently incubated with 293T/ACE2 cells^3^ (for all WT neutralization assays) or HT1080/ACE2.cl14 cells (for all mutant panels and variant neutralization assays)^13^ for 48 h, after which cells were washed with PBS and lysed with Luciferase Cell Culture Lysis 5× reagent (Promega). Nanoluc luciferase activity in lysates was measured using the Nano-Glo Luciferase Assay System (Promega) with the Glomax Navigator (Promega). Relative luminescence units were normalized to those derived from cells infected with SARS-CoV-2-pseudotyped virus in the absence of plasma or monoclonal antibodies. The NT_50_ values for plasma or IC_50_ and 90% inhibitory concentrations for monoclonal antibodies were determined using four-parameter nonlinear regression (least-squares regression method without weighting; constraints: top = 1, bottom = 0) (GraphPad Prism).

### Biotinylation of viral protein for use in flow cytometry

Purified and Avi-tagged SARS-CoV-2 RBD or SARS-CoV-2 RBD K417N/E484K/N501Y mutant was biotinylated using the Biotin-Protein Ligase-BIRA kit according to the manufacturer’s instructions (Avidity) as described before^3^. Ovalbumin (Sigma, A5503-1G) was biotinylated using the EZ-Link Sulfo-NHS-LC-Biotinylation kit according to the manufacturer’s instructions (Thermo Scientific). Biotinylated ovalbumin was conjugated to streptavidin-BV711 (BD Biosciences, 563262), and RBD was conjugated to streptavidin-PE (BD Biosciences, 554061) and streptavidin-AF647 (BioLegend, 405237)^3^.

### Flow cytometry and single-cell sorting

Single-cell sorting by flow cytometry was described previously^3^. In brief, PBMCs were enriched for B cells by negative selection using a pan-B cell isolation kit according to the manufacturer’s instructions (Miltenyi Biotec, 130-101-638). The enriched B cells were incubated in FACS buffer (1× PBS, 2% FCS, 1 mM EDTA) with the anti-human antibodies (all at a 1:200 dilution) anti-CD20-PECy7 (BD Biosciences, 335793), anti-CD3-APC-eFluro 780 (Invitrogen, 47-0037-41), anti-CD8-APC-eFluor 780 (Invitrogen, 47-0086-42), anti-CD16-APC-eFluor 780 (Invitrogen, 47-0168-41) and anti-CD14-APC-eFluor 780 (Invitrogen, 47-0149-42), as well as Zombie NIR (BioLegend, 423105) and fluorophore-labelled RBD and ovalbumin (Ova) for 30 min on ice. Single CD3^–^CD8^–^CD14^–^CD16^–^CD20^+^Ova^−^RBD-PE^+^RBD-AF647^+^ B cells were sorted into individual wells of 96-well plates containing 4 μl of lysis buffer (0.5× PBS, 10 mM dithiothreitol, 3,000 U ml^–1^ RNasin Ribonuclease Inhibitors (Promega, N2615)) per well using a FACSAria III and FACSDiva software (Becton Dickinson) for acquisition and FlowJo software for analysis. The sorted cells were frozen on dry ice and then stored at −80 °C or immediately used for subsequent RNA reverse transcription. For plasmablast single-cell sorting, in addition to the above antibodies, B cells were also stained with anti-CD19-BV605 (BioLegend, 302244) and single CD3^–^CD8^–^CD14^–^CD16^–^CD19^+^CD20^–^Ova^–^RBD-PE^+^RBD-AF647^+^ plasmablasts were sorted as described above. For B cell phenotype analysis, in addition to the above antibodies, B cells were also stained with the following anti-human antibodies (all at a 1:200 dilution): anti-IgD-BV421 (BioLegend, 348226), anti-CD27-FITC (BD Biosciences, 555440), anti-CD19-BV605 (BioLegend, 302244), anti-CD71-PerCP-Cy5.5 (BioLegend, 334114), anti-IgG-PECF594 (BD Biosciences, 562538), anti-IgM-AF700 (BioLegend, 314538) and anti-IgA-Viogreen (Miltenyi Biotec, 130-113-481).

### Antibody sequencing, cloning and expression

Antibodies were identified and sequenced as described previously^3,55^. In brief, RNA from single cells was reverse transcribed (SuperScript III Reverse Transcriptase, Invitrogen, 18080-044), and the cDNA was stored at −20 °C or used for subsequent amplification of the variable *IGH*, *IGL* and *IGK* genes by nested PCR and Sanger sequencing. Sequence analysis was performed using MacVector. Amplicons from the first PCR reaction were used as templates for sequence- and ligation-independent cloning into antibody expression vectors. Recombinant monoclonal antibodies were produced and purified as previously described^3^.

### Biolayer interferometry

BLI assays were performed as previously described^3^. In brief, we used the Octet Red instrument (ForteBio) at 30 °C with shaking at 1,000 r.p.m. Affinity measurement of anti-SARS-CoV-2 IgG binding was corrected by subtracting the signal obtained from traces performed with IgGs in the absence of WT RBD. Kinetic analysis using protein A biosensor (ForteBio, 18-5010) was performed as follows: (1) baseline: immersion for 60 s in buffer; (2) loading: immersion for 200 s in a solution with IgGs at 10 μg ml^–1^; (3) baseline: immersion for 200 s in buffer; (4) association: immersion for 300 s in solution with WT RBD at 20, 10 or 5 μg ml^–1^; (5) dissociation: immersion for 600 s in buffer. Curve fitting was performed using a fast 1:1 binding model and the data analysis software from ForteBio. Mean equilibrium dissociation constants (*K*_d_) were determined by averaging all binding curves that matched the theoretical fit with an *R*^2^ value ≥0.8.

### Computational analyses of antibody sequences

Antibody sequences were trimmed on the basis of quality and annotated using Igblastn v.1.14 with the IMGT domain delineation system. Annotation was performed systematically using Change-O toolkit v.0.4.540 (ref. ^56^). Heavy and light chains derived from the same cell were paired, and clonotypes were assigned on the basis of their V and J genes using in-house R and Perl scripts. All scripts and the data used to process antibody sequences are publicly available on GitHub (https://github.com/stratust/igpipeline/tree/igpipeline2_timepoint_v2).

The frequency distributions of human V genes in anti-SARS-CoV-2 antibodies from this study were compared with 131,284,220 IgH and IgL sequences generated in ref. ^57^ and downloaded from cAb-Rep^58^, a database of shared human B cell antigen receptor (BCR) clonotypes available at https://cab-rep.c2b2.columbia.edu/. On the basis of the 112 distinct V genes that made up the 7,936 analysed sequences from the immunoglobulin repertoire of the 11 participants present in this study, we selected the IgH and IgL sequences from the database that were partially encoded by the same V genes and counted them according to the constant region. The frequencies shown in Extended Data Fig. 4 are relative to the source and isotype analysed. We used the two-sided binomial test to check whether the number of sequences belonging to a specific *IGHV* or *IGLV* gene in the repertoire was different according to the frequency of the same IgV gene in the database. Adjusted *P* values were calculated using the false discovery rate (FDR) correction. Significant differences are denoted with asterisks.

Nucleotide somatic hypermutation and CDR3 length were determined using in-house R and Perl scripts. For somatic hypermutations, *IGHV* and *IGLV* nucleotide sequences were aligned against the closest germline sequences using Igblastn and the number of differences was considered to correspond to nucleotide mutations. The average number of mutations for V genes was calculated by dividing the sum of all nucleotide mutations across all participants by the number of sequences used for the analysis.

### Data presentation

Figures were arranged in Adobe Illustrator 2020.

### Reporting summary

Further information on research design is available in the Nature Research Reporting Summary linked to this paper.

## Online content

Any methods, additional references, Nature Research reporting summaries, source data, extended data, supplementary information, acknowledgements, peer review information; details of author contributions and competing interests; and statements of data and code availability are available at 10.1038/s41586-021-04060-7.

### Supplementary information

Reporting Summary

Supplementary Table 1 Summary of cohort characteristics.

Supplementary Table 2 Summary of individual participant characteristics.

Supplementary Table 3 Half-maximal neutralization titres (NT_50_ values) of plasma against different mutant SARS-CoV-2 pseudoviruses, for paired samples from participants 1.3 and 5 months after the second vaccination.

Supplementary Table 4 Sequences of anti-SARS-CoV-2 RBD antibodies derived from single cells.

Supplementary Table 5 Sequences, RBD binding (EC_50_) and neutralization (IC_50_) of cloned recombinant antibodies.

Supplementary Table 6 RBD binding (EC_50_) and neutralization (IC_50_) of shared clones of recombinant antibodies isolated after vaccination.

Supplementary Table 7 Neutralization activity of recombinant antibodies against mutant SARS-CoV-2 pseudoviruses, in antibodies isolated after the prime and 1.3 months after the boost vaccination.

Supplementary Table 8 Neutralization activity of recombinant antibodies against mutant SARS-CoV-2 pseudoviruses, in clonal pairs of antibodies isolated at 1.3 and 6.2 months after infection, as well as 1.3 and 5 months after the second vaccination.

## Figures and Tables

**Extended Data Table 1:**
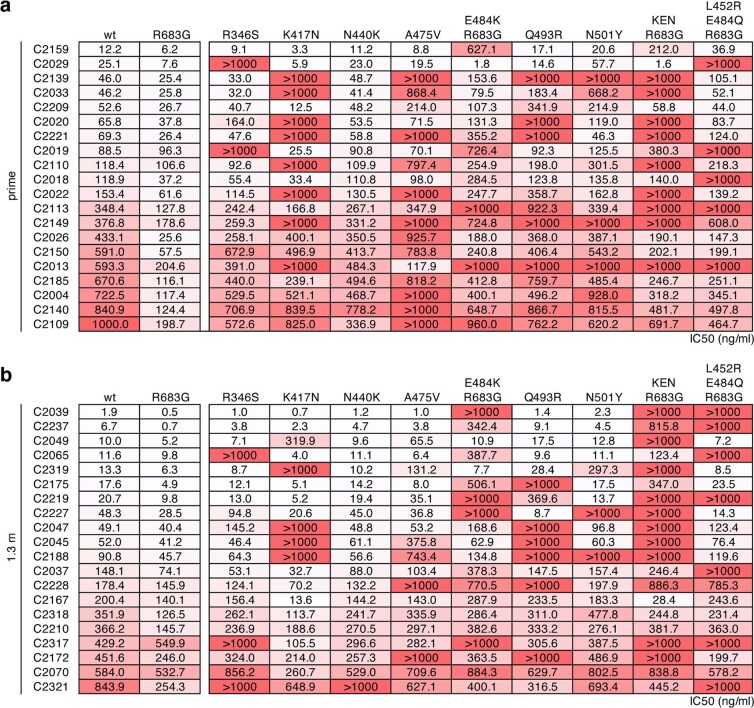
Breadth of anti-SARS-CoV-2 RBD antibodies elicited after prime and 2 doses of vaccination

**a-b**, IC_50_ values for n=40 neutralizing antibodies isolated after prime **(a)** or 1.3 months post-boost **(b)** against indicated mutant SARS-CoV-2 pseudoviruses. Color gradient indicates IC_50_ values ranging from 0 (white) to 1000 ng/ml (red).

## Data Availability

Data are provided in Supplementary Tables 1–8. The raw sequencing data and computer scripts associated with Fig. 2 and Extended Data Fig. 3 have been deposited at GitHub (https://github.com/stratust/igpipeline/tree/igpipeline2_timepoint_v2). This study also uses data from 10.5061/dryad.35ks2, the Protein Data Bank (6VYB and 6NB6), cAb-Rep (https://cab-rep.c2b2.columbia.edu/), the Sequence Read Archive (accession SRP010970) and ref. ^57^ (10.1038/s41586-019-0934-8).

## Code availability

Computer code to process the antibody sequences is available at GitHub (https://github.com/stratust/igpipeline/tree/igpipeline2_timepoint_v2).
